# Electroanalytical Sensing of Bromides Using Radiolytically Synthesized Silver Nanoparticle Electrocatalysts

**DOI:** 10.1155/2017/2028417

**Published:** 2017-10-17

**Authors:** Jadranka Milikić, Ivan Stoševski, Jelena Krstić, Zorica Kačarević-Popović, Šćepan Miljanić, Biljana Šljukić

**Affiliations:** ^1^Faculty of Physical Chemistry, University of Belgrade, Studentski trg 12–16, 11158 Belgrade, Serbia; ^2^Vinča Institute of Nuclear Sciences, University of Belgrade, P.O. Box 522, 11001 Belgrade, Serbia

## Abstract

Monitoring bromides (Br^−^) is of crucial importance since bromates, potential human carcinogens, are formed during ozonation of water containing bromides in concentrations >100 *μ*g L^−1^. Within this study, silver (Ag) and four carbon-supported Ag catalysts were synthesized by the *γ*-radiation method and their morphology and structure examined using transmission electron microscopy, X–ray diffraction, and UV-Vis analysis. The nanocatalysts were tested for Br^−^ sensing in aqueous media using cyclic voltammetry. All five Ag materials exhibited electroactivity for sensing of Br^−^ ions, with pure Ag catalyst giving the best response to Br^−^ ions presence in terms of the lowest limit of detection. Sensing of bromides was also explored in tap water after addition of bromides suggesting that herein prepared catalysts could be used for bromides detection in real samples. Furthermore, sensing of other halogen ions, namely, chlorides and iodides, was examined, and response due to chloride presence was recorded.

## 1. Introduction

**Figure 1:**
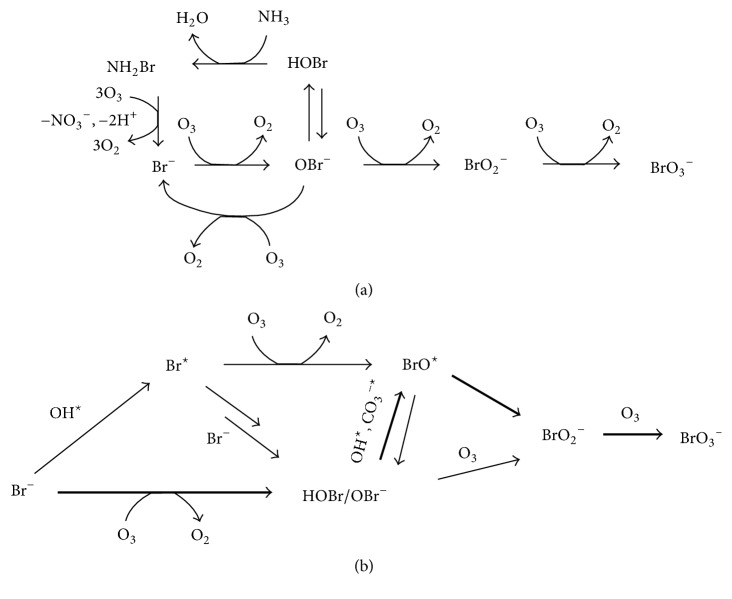
Possible pathways of bromate formation during ozonation of bromide-containing waters: oxidation with (a) O_3_ and (b) OH^∙^ [1].

According to the UN-Water Global Analysis and Assessment of Sanitation and Drinking Water (GLAAS) 2017 report, nearly two billion people use drinking water from sources polluted with faeces with over 500,000 diarrhoeal deaths per year. Ozone is a powerful disinfectant which can disarm even those resilient pathogenic microorganisms that common disinfectants such as chlorine and chlorine dioxide cannot. Still, high ozone exposure is necessary to neutralize these microorganisms, resulting in generation of undesirable disinfection by-products [1]. Thus, during ozonation of bromide (Br^−^)-containing waters, different bromoorganic by-products are generated in the reaction of hypobromous acid (product of the reaction of Br^−^ and O_3_) with natural organic matter. Concentrations of these bromoorganic compounds are typically far below the drinking water standards. However, the main by-product of concern is bromate (BrO_3_^−^), generated by the oxidation of bromide by both O_3_ and OH^∙^, including several pathways and up to six oxidation states of bromine, Figure 1.

Bromate, unlike many other organic by-products, is not degraded in biological filters applied upon the ozonation process. Bromate is known to be the genotoxic carcinogen inducing, for instance, renal cell tumor in rats. It is believed that bromate toxic effects arise from the elevated levels of lipid peroxides (LPO) or from oxygen radicals formed from LPO and to induce DNA damage.

Thereafter, monitoring the level of halogen ions in water is nowadays of great importance for the protection of human health. Methods so far used or suggested for the detection of bromide and other halogens in water include neutron activation analysis, inductively coupled plasma–mass spectrometry, ion and liquid chromatography, and fluorescence spectroscopy [2–7]. These techniques often require complex procedures and expensive equipment.

On the other hand, electrochemical methods for detection of halogen ions offer benefits of low cost, simplicity, and rapidity [8–11]. Silver (Ag) and Ag-based materials have been investigated as sensing electrode for these purposes [8, 12, 13]. Ag nanoparticles (NPs) can be synthesized in a variety of ways, with the most frequently employed being chemical-reduction methods. These methods involve reduction of Ag^+^ ions in aqueous solution using different reducing agents that can be toxic or non-environmentally friendly and the procedure comprises several time-consuming steps, including addition of reducing agent in the solution, filtering, washing, and drying.

Radiation methods of synthesis have several advantages over the other methods: rapidity, low operational costs, no reducing agent required, easier control of the synthesis parameters, absence of unnecessary synthesis products, convenience for large scale use, and environmental friendliness. A major advantage of the radiation synthesis of metal NPs is a homogenous reduction of metal ions throughout the solution. Radiation methods have already been used for preparation of Ag NPs for biomedical applications. In this method, Ag^+^ ions were reduced by radiolytic products of water, where polymers, such as the poly(vinyl alcohol) and the chitosan, act as stabilizers for controlling the size of Ag NPs [14]. We have for the first time synthesized Ag NPs by *γ*–radiation method and investigated them as electrocatalysts for the oxygen reduction and borohydride oxidation reactions [15]. With respect to that research, this paper investigates similarly prepared Ag NPs with a focus on a different application.

The goal of this research is to investigate the potential application of Ag nanocatalysts, synthesized by the *γ*-radiation method, as electrode materials for detection of the halogen ions (Br^−^, Cl^−^, and I^−^). As mentioned above, the radiation method, used in this work, enables direct preparation of the sensing electrode in a simple and a much faster way than a conventional approach, avoiding filtering, washing, and drying steps. The polymer used for controlling the Ag NPs size during the radiation procedure (synthesis of the Ag NPs) can later be used as the active material binder. Thus, in our approach, the Ag NP ink was prepared from the precursor in one-step radiation process. Prepared Ag NPs electrocatalysts were tested for detection of bromides, chlorides, and iodides using cyclic voltammetry.

## 2. Experimental

### 2.1. Materials

Poly(vinyl alcohol) (PVA) with mean molecular weight *M*_*w*_ of 72 kDa (degree of hydrolysis of 99%), silver nitrate (AgNO_3_), glutaraldehyde (GA, 25 wt.% aqueous solution), and 2-propanol ((CH_3_)_2_CHOH) were purchased from Merck, while argon gas was obtained from Messer Tehnogas, Serbia. The medium molecular weight chitosan (75–85% degree of deacetylation, *M*_*w*_ from 190 to 310 kDa) and potassium sulfate (K_2_SO4), bromide (KBr), chloride (KCl), and iodide (KI) were supplied by Sigma-Aldrich. Carbon black, Vulcan XC 72R, was produced by Cabot Co. The chemicals were used as received, without additional purification. Water from Millipore Milli-Q system was used in all experiments.

### 2.2. Electrocatalyst Preparation

**Figure 2:**
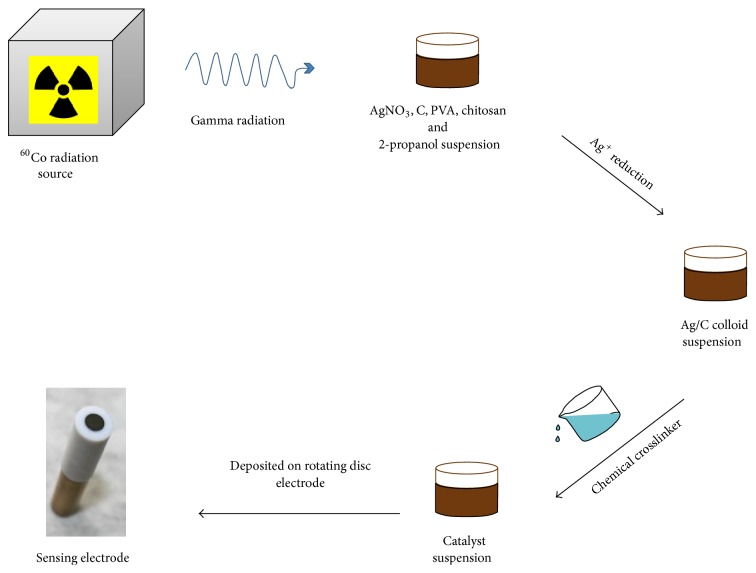
Schematic representation of catalysts and working electrodes preparation procedure.

Both types of Ag NPs, nonsupported and supported on the carbon substrate (Ag/C), were synthesized by the *γ*–radiation reduction method using PVA and PVA/chitosan as a stabilizer (Figure 2), as described in the authors' previous paper [15]. In brief, five aqueous solutions containing polymer (weight ratio of PVA to CS was 3 : 1), AgNO_3_, and (CH_3_)_2_CHOH were prepared. All solutions had constant concentration of AgNO_3_ (0.4 M) and (CH_3_)_2_CHOH (0.2 M), while concentration of polymer varied (2, 4, and 6 wt.%). Different amounts of Vulcan XC 72R were added to the four out of five solutions (Table 1) and the obtained dispersions were homogenized ultrasonically. Four dispersions and one solution without carbon were then deaerated with argon for 20 min in an air–tight glass vessel, and after that they were exposed to *γ*–rays (^60^Co source) at the temperature of 22°C. The absorbed dose and the dose rate were 700 kGy and 12 kGy h^−1^, respectively.

### 2.3. Characterization

Transmission electron microscopy (TEM) analysis was done using the JEOL JEM–1400Plus device, while the X–ray diffraction (XRD) analysis was done using Bruker D8 Advance Diffractometer (Cu K_*α*1_ radiation, *λ* = 0.1541 nm). Optical characterization of the as-prepared Ag NPs was carried out using Thermo Scientific Evolution 600 UV-Vis spectrophotometer in the 300–800 nm wavelength range.

### 2.4. Electrochemical Study

The working electrode was prepared as follows: 0.5 wt.% GA solution was added to each of the obtained catalyst dispersions (molar ratio of GA to polymer was 8.2 : 1), after which the dispersions were diluted and the appropriate volume of 1 M HCl was added, in order to obtain HCl concentration of 0.1 M in the final dispersion. 10 *μ*L of this dispersion was dropped on the glassy carbon electrode (GCE, 0.196 cm^2^, Pine Instruments Co.) resulting in Ag loading on the electrode of 20 *μ*g cm^−2^. Crosslinking of the polymer with GA was achieved by HCl during the drying under the nitrogen atmosphere, and the gel that was formed acted as a binder. In order to wash out the Cl^−^ ions from the electrodes, they were immersed for 10 minutes in deionized water.

Electrochemical characterization of the five Ag catalysts was done by cyclic voltammetry (CV) using a Gamry PCI4/750 potentiostat/galvanostat. For all electrochemical measurements within this study, catalyst-coated GCE served as working electrode; Pt foil was employed as the counter electrode and a saturated calomel electrode (SCE, Radiometer Analytical) as reference. All potentials are reported relative to the SCE. CVs were recorded in 0.1 M K_2_SO_4_ solution as supporting electrolyte. Br^−^ ions detection was done by CV measurements in bromide solution in 0.1 M K_2_SO_4_ in the potential range from −0.9 V to 0.5 V at scan rate of 100 mVs^−1^. Cl^−^ and I^−^ ions detection was assessed under the same experimental conditions.

## 3. Results and Discussion

### 3.1. Characterization of the Ag and Ag/C Catalysts

The radiation-induced reduction of Ag^+^ starts with the radiolysis of water and formation of hydrated electrons, hydrogen (H^*∙*^) and hydroxyl radicals (OH^∙^). Hydrated electrons and H^*∙*^ radicals reduce Ag^+^ to Ag^0^, which further dimerize when they encounter or associate with Ag^+^ and progressively grow yielding the formation of metal clusters and particles. The detailed mechanism of Ag NP formation by radiation methods has been explained by Belloni [16]. Since the OH^∙^ radicals can oxidize the metal atoms into a higher oxidation state and thus counterbalance the reduction, (CH_3_)_2_CHOH is used as a scavenger to convert them to 2–propanol radicals (^*∙*^(CH_3_)_2_CHOH), which further act as strong reducing species. It should be mentioned that the Ag NP dispersions are stable at room temperature for several days. However, at lower temperatures (around 7°C), the pure Ag dispersion (101) can withstand more than a year without precipitation of Ag.

**Figure 3:**
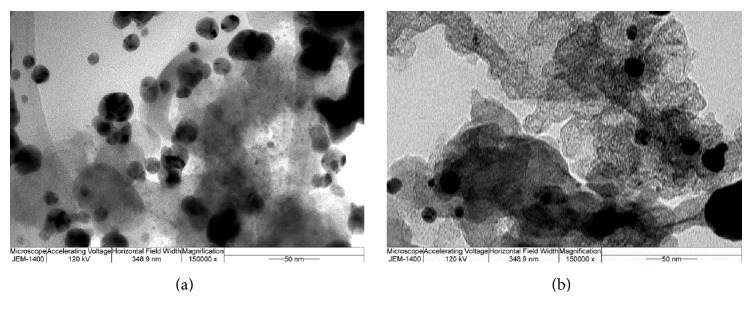
Representative TEM micrographs of Ag/C catalyst obtained from the colloid suspensions 214 (a) and 116 (b).

The detailed XRD and TEM analysis of the Ag NPs obtained by the *γ*–radiation method was reported in our previous publication [15], in which different Ag/C samples were investigated as electrocatalysts for the oxygen reduction and borohydride oxidation reaction. In brief, TEM analysis of Ag/C samples has shown that an average particle size of Ag NPs is between 14 nm and 18 nm and that there is no big difference in average particle diameters between samples. The TEM micrographs of Ag samples obtained from the colloid suspensions 214 and 116 are presented in Figure 3. It was observed that number of smaller particles is notably higher than the number of larger ones. Furthermore, bimodal particle size distributions are seen for all studied electrocatalysts, most likely due to the high concentration of Ag compared to that of stabilizer and possibly due to the interaction of Ag NPs with C affecting the NPs stabilization [15]. XRD analysis (not shown) revealed the formation of face centered cubic (FCC) crystal structure of bulk metallic Ag in case of Ag, Ag_214_/C, Ag_114_/C, and Ag_116_/C, and mixture of metallic Ag and Ag oxide NPs in case of Ag_112_/C [15]. It was observed that higher amount of C and Ag : polymer ratio different than 1 : 1 led to disturbance of the crystal structure. Additionally, crystallite size, texture coefficient (*C*_txt_), interplanar spacing between atoms (*d*), and lattice parameter (*a*) of Ag, Ag_214_/C, Ag_114_/C, and Ag_116_/C were determined from the XRD pattern data. Thus, crystallite size of the samples determined by XRD was found to be from 14.6 nm to 20.0 nm, which is in good agreement with the crystallite size evaluated by TEM. Obtained values of *C*_txt_ for (111), (200), (220), and (311) planes were evaluated to be in the range from 0.55 to 1.05, with these values indicating planes with no preferential growth orientation. Δ*d*/*d*_0_^(111)^ ranging from −0.0001 to −0.0018 and Δ*a*/*a*_0_^sr^ values ranging from −0.0008 to −0.0022 confirmed formation of bulk FCC Ag structure. The lattice parameters were found to be lower than the bulk lattice constant indicating presence of strain and stress that could further lead to the change in the bond length and to the change of energy levels of the bonding electrons [15, 17].

**Figure 4:**
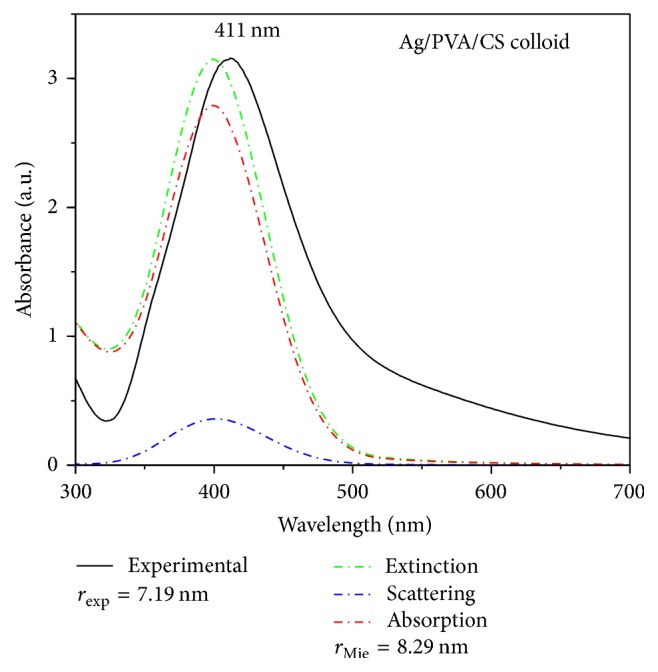
UV-Vis absorption spectra of Ag_101_ colloid suspension. The solid line corresponds to the experimental spectrum and the dashed lines correspond to the calculated simulation spectra using the Mie model.

UV-Vis spectrum of Ag/polymer (Ag_101_) colloid is shown in Figure 4 (solid line). For nanoparticles smaller than 20 nm, the average radius of the nanoparticles can be determined by the full width at half maximum (FWHM) of their corresponding SPR band using the relation

(1) $$\begin{array}{c} r_{exp}=\frac{V_{f}}{\Delta \omega_{1/2}}, \end{array}$$

where *r*_exp_ is the particle radius, *V*_*f*_ is the Fermi velocity of the metal, and Δ*ω*_1/2_ is the FWHM for the SPR band in units of angular frequency [18]. The calculated experimental average particle diameters of as-prepared Ag NPs are given in Table 2. Taking into account the calculated values for the Ag NPs radii, the corresponding theoretical optical extinction spectra are simulated using a computer program “MiePlot v.3.4” [19], whose algorithm is based on the Mie's theory (Figure 4, dashed lines) [20].

The calculated values of average Ag_101_ NPs diameters obtained by the fitting to experimental spectra are given in Table 2. Size of Ag NPs in the colloid sample Ag_214_ has been determined as well (Table 2), while this was not possible for other dispersions due to a large amount of carbon. As it can be seen in Table 2, the average sizes of Ag NPs in the colloid samples 101 and 214 determined by UV-Vis, XRD, and TEM analysis are in good correlation. Considering the experimentally determined diameters and that Ag NPs are ideal spheres, the surface area (SA) of Ag NPs was calculated using the equation SA = 6/*ρD*, where *ρ* is the theoretical density of Ag (10.5 g cm^−3^) and* D* is the diameter of Ag NPs.

**Figure 5:**
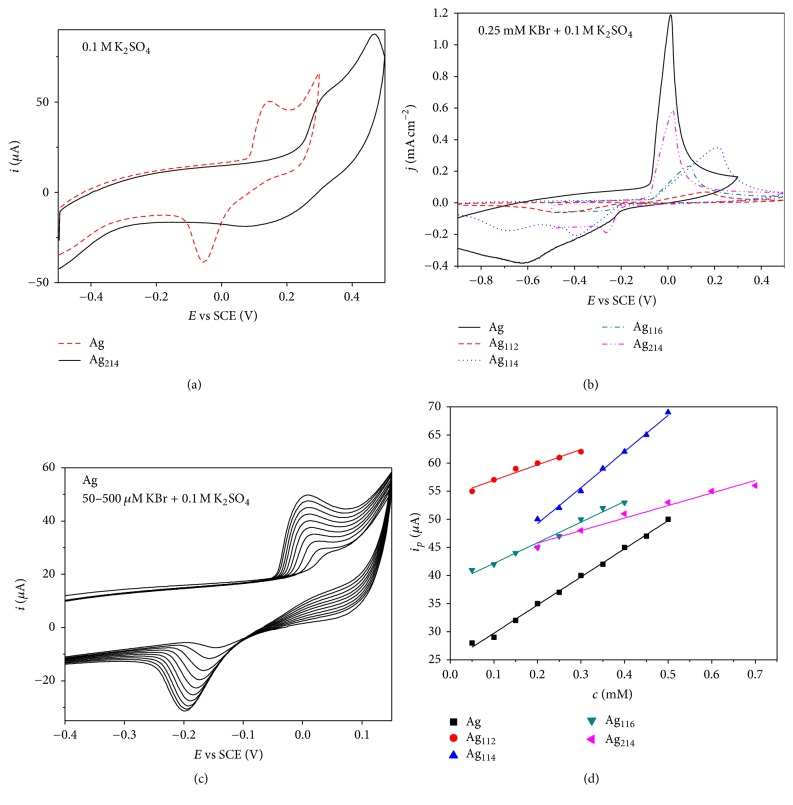
(a) CVs of Ag and Ag_214_/C catalyst in 0.1 M K_2_SO_4_ solution at scan rate of 100 mV s^−1^; (b) CVs of all five catalysts in 0.25 mM KBr + 0.1 M K_2_SO_4_ at scan rate of 100 mVs^−1^; (c) CVs of Ag catalyst in 0.1 M K_2_SO_4_ as supporting electrolyte with increasing KBr concentration from 0.05 to 0.5 mM at scan rate of 10 mVs^−1^; (d) oxidation peak current as a function of KBr concentration for all five catalysts.

Electrochemical characterization was done in 0.1 M K_2_SO_4_ at scan rate of 100 mV s^−1^.  Figure 5(a) illustrates the electrochemical characterization of pure Ag catalyst where Ag oxidation to Ag_2_O is observed as peak at ca. 0.15 V. On the cathodic scan, pronounced oxide reduction peak could be seen at ca. −0.05 V in agreement with the literature data [8]. Qualitatively similar CVs were obtained for other Ag electrocatalysts; namely, distinctly defined Ag oxidation peaks were observed at potentials above 0.25 V with corresponding oxides reduction peak in the potential range from 0.15 V to 0 V (Figure 5(a) shows CV of Ag_214_). Charge under the reduction peaks was used to evaluate the electrochemical surface area (ECSA), assuming that charge associated with one Ag oxide monolayer is 2.10 C m^−2^ [21]. These ECSA were used to calculate specific current densities.

### 3.2. Sensing of Bromides

Subsequently, possibility of using prepared Ag and Ag/C catalysts for electroanalytical sensing of bromides was examined. Therefore, CVs of all five Ag materials were recorded in 0.25 mM KBr + 0.1 M K_2_SO_4_ in the potential range from −1 V to 0.5 V at scan rate of 100 mVs^−1^ (Figure 5(b)). All five catalysts gave well defined oxidation peak in the potential range of 0–0.2 V evidencing formation of silver bromide (AgBr). Formed AgBr was reduced on the reverse scan with the reduction peak seen at potentials lower than ca. −0.2 V. The principle of bromide (as well as other halogen ions) sensing by Ag electrodes can be described by [26]

(2) $$\begin{array}{c} Ag+Br^{-}⟶AgBr+e^{-} \end{array}$$

Pure Ag catalyst was observed to give the highest peak current density (*j*) value (1.19 mA cm^−2^), followed by Ag_214_/C (0.59 mA cm^−2^), Ag_114_/C (0.36 mA cm^−2^), Ag_116_/C (0.23 mA cm^−2^), and Ag_112_/C (0.07 mA cm^−2^). It is worth noting that these peak current/current density values are one order of magnitude higher than the current values obtained by different Ag NPs reported in the literature [8]. Although metal nanocatalysts are usually supported on carbon materials in order to increase their active surface area and to improve their utilization, herein nonsupported Ag NP outperformed the supported ones. Differences in the activity of the five studied electrocatalysts for Br^−^ sensing can be attributed to the differences in their particle size, surface structure, and C and polymer percentage. The highest peak current density observed for Ag and then Ag_214_/C can be correlated with the smaller particle size of these two electrocatalysts and consequently to their higher specific surface area. Furthermore, it can be observed that the lower peak current densities are observed for electrocatalysts containing higher percentage of C (Ag_114_/C, Ag_116_/C, and Ag_112_/C), suggesting that C hinders access of Br^−^ ions to Ag surface. Surface structure also plays significant role in the catalytic activity of Ag-based materials as it was shown that different Ag surface planes catalyze electrochemical reactions at different rate [15].

Subsequently, CVs of all five Ag catalysts were recorded in 0.1 M K_2_SO_4_ solution increasing the KBr concentration with 50 *μ*M additions of KBr to 0.1 M K_2_SO_4_, except in the case of Ag_214_/C catalyst where 100 *µ*M additions were made (Figure 5(c) shows the corresponding CVs of pure Ag). Increase of peak current (*i*_*p*_) could be observed with increase of KBr concentration (*c*), with *i*_*p*_ versus* c* dependence resulting in straight lines with correlation coefficients in the 0.96008–0.99648 range, Figure 5(d).

The limit of detection (LOD) of bromide at Ag and Ag/C catalysts was evaluated using the 3-sigma method

(3) $$\begin{array}{c} LOD=\frac{3\sigma }{b}, \end{array}$$

where *σ* is the standard deviation of the *y* coordinates from the line of best fit and* b* the slope of the same line. The lowest limit of bromides detection of 18 *µ*M was evaluated with pure Ag catalyst. Ag_114_/C and Ag_116_/C gave similar LODs of 36 and 40 *μ*M, respectively, followed by Ag_112_/C that gave LOD of 53 *μ*M, while the highest LOD of 102 *μ*M was obtained using Ag_214_/C, Table 3. Good reproducibility of the catalysts performance was observed with standard deviation (10 measurements) in the 4.8–8.3% range. It should be noted that higher sensitivity with Ag electrodes studied herein could be obtained using more sensitive electroanalytical methods (such as square wave voltammetry, differential pulse voltammetry, and chronoamperometry) and with optimisation of operational parameters. Still, the LOD value obtained at pure Ag catalyst was somewhat higher or comparable with LODs obtained using different Ag-based catalysts for bromide sensing reported in the literature (LODs in 1.2–22 *μ*M range) [23–29]. For example, Compton et al. reported LOD for bromides of 3 *μ*M at AgNPs-GC-epoxy composite electrode and of 22 *μ*M at GCM-Ag/MWCNT/GCE (Glassy Carbon Metal, Multi Wall Carbon Nanotube, Glassy Carbon Electrode) [8, 25]. Furthermore, Binghui et al. reported LODs for bromide between 0.023 and 2.0 *μ*g L^−1^ using ion chromatography [6].

### 3.3. Sensing of Chlorides and Iodides

**Figure 6:**
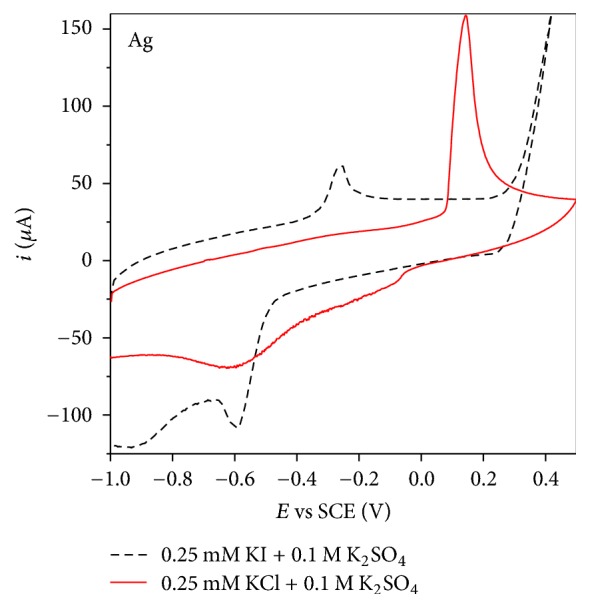
CVs of pure Ag catalyst in 0.25 mM KI (- - -) and 0.25 mM KCl (—) in 0.1 M K_2_SO_4_ as supporting electrolyte recorded at scan rate of 100 mV s^−1^.

As pure Ag catalyst gave the lowest LOD for bromides, it was further tested for sensing of other halogen ions, namely, chlorides and iodides. For that purpose, CV of pure Ag catalyst was recorded in 0.25 mM KCl solution under the same experimental conditions as in the case of bromides detection, Figure 6. A clear oxidation peak evidencing formation of silver chloride (AgCl) could be seen at ca. 0.15 V, indicating potential application of Ag catalyst for chloride sensing. Furthermore, CVs of Ag catalyst were recorded increasing the KCl concentration from 50 to 500 *μ*M. Dependence of the oxidation peak current on KCl concentration resulted in a straight line with correlation factor of 0.98678, and data from that line were used for evaluation of the limit of chloride detection using Ag catalyst. Thus, LOD of chloride was determined to be 51.3 *μ*M with standard deviation (10 measurements) of 6.9%. Sánchez-Polo et al. reported LOD for bromide and chloride ions of 3 and 1 *μ*g L^−1^, respectively, by ion chromatography [30].

Contrary to CVs of Ag catalyst recorded in 0.25 mM KBr and 0.25 mM KCl solutions, where well defined oxidation peaks were observed, only a vague peak at ca. −0.25 V could be seen at the CV recorded in 0.25 mM KI solution. Increase of KI concentration in the 50 to 500 *μ*M range did not result in the noteworthy increase of the oxidation peak current. Thus, due to the poor signal, limit of iodide detection using Ag catalyst could not be determined.

### 3.4. Sensing of Bromides in Real Samples

**Figure 7:**
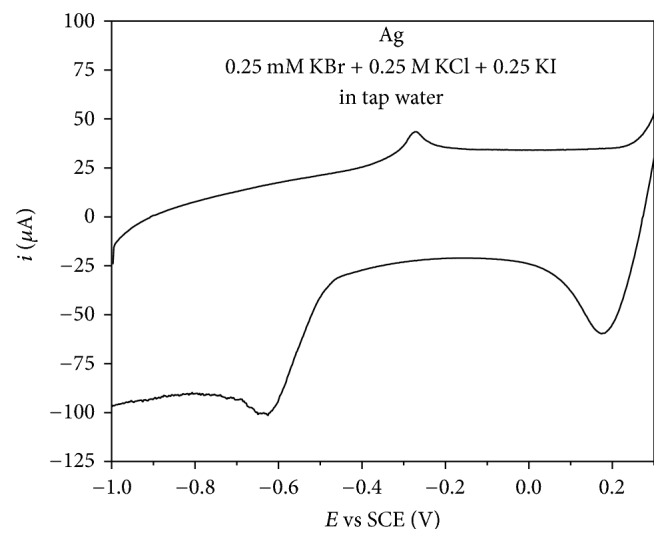
CV of Ag catalyst in 0.25 mM KBr + 0.25 mM KCl + 0.25 mM KI in tap water recorded at scan rate of 100 mV s^−1^.

Finally, possibility of using Ag catalyst for sensing of bromides in real sample was explored by recording CV of Ag catalyst in tap water after addition of 0.25 mM KBr + 0.25 mM KCl + 0.25 mM KI without any pretreatment of the sample. No peak was observed at CV recorded in tap water prior to halides addition. Conversely, two peaks could be observed, a peak at ca. 0.18 V corresponding to bromide presence and a peak at ca. −0.6 V corresponding to chloride presence, Figure 7. Again, no peak due to presence of iodides could be seen. Clear separation of two peaks confirms that Ag could be used for bromides sensing in real samples in the presence of interferents as well.

The results indicate that Ag and Ag/C catalysts prepared within this study could be used as sensing electrode materials for detection of different halogen ions, namely, Br^−^ and Cl^−^ ions. Among studied catalysts, pure Ag NPs showed the best performance for halogen ions sensing.

## 4. Conclusions

Ag NPs and carbon-supported Ag NPs of ca. 14–18 nm diameter were prepared using *γ*-radiation method and evaluated as sensing electrodes for bromide detection. Limit of bromides detection was evaluated using the 3-sigma method. Pure Ag electrocatalyst gave the best results as evidenced by the lowest LOD of 18 *μ*M. Furthermore, this catalyst was shown to be active for chloride detection as well. Finally, bromide sensing was assessed in real sample and the catalyst gave a clear response to bromides presence suggesting catalysts' potential application for sensing of bromides in real samples.

## Figures and Tables

**Table 1:** Weight ratios of Ag, C, and polymer in the initial colloid suspensions.

Colloid suspension number	Ag : C : Polymer
101	1 : 0 : 1
214	1 : 0.4 : 1
116	1 : 1 : 2
114	1 : 1 : 1
112	1 : 1 : 0.5

**Table 2:** Ag NP sizes evaluated using UV-Vis spectroscopy and using Mie's theory.

Sample	FWHM (Δ*λ*) (nm)	Average diameter (nm)		Surface area (m^2^ g^−1^)
		UV/Vis	Mie	
101	97.51	14.2	16.6	40.2
214	110.50	16.3	17.7	35.1

**Table 3:** Comparison of performance of different electrodes for bromide sensing.

Catalyst	Electrolyte	Peak current/*μ*A	Peak potential/V	LOD
Ag (present catalyst)	0.25 mM KBr + 0.1 M K_2_SO_4_	310	0.01	18 *μ*M
Ag_112_/C (present catalyst)	0.25 mM KBr + 0.1 M K_2_SO_4_	446	0.16	53 *μ*M
Ag_114_/C (present catalyst)	0.25 mM KBr + 0.1 M K_2_SO_4_	704	0.21	36 *μ*M
Ag_116_/C (present catalyst)	0.25 mM KBr + 0.1 M K_2_SO_4_	390	0.09	40 *μ*M
Ag_214_/C (present catalyst)	0.25 mM KBr + 0.1 M K_2_SO_4_	317	0.02	102 *μ*M
AgNP-GC–BPPG^*∗*^ [8]	0.16 mM KBr + 0.1 M K_2_SO_4_	≈15	≈0.14	3 *μ*A
Ag/Au/PdNP-GC–BPPG^*∗*^ [8]	0.16 mM KBr + 0.1 M K_2_SO_4_	≈25	≈0.18	/
Ag electrode [22]	2 mM bromide ions in pH 7 phosphate buffer	/	≈0	5 mM
Hg (II) complex^*∗∗*^ [23]	/	/	/	4 *μ*M
MWNTs-chitosan modified GCE^*∗∗∗*^ [24]	7.2 · 10^−6^ gmL^−1^ bromide ions in pH 1.8 H_2_SO_4_ solution	≈78	0.71	9.6·10^−8^ g ml^−1^
GCM-Ag/MWCNT/GC^*∗∗∗∗*^ [25]	5 mM bromide ions in 0.1 M phosphate buffer	≈750	≈0.1	22 *μ*M

^*∗*^NP-GC–BPPG: nanoparticles on glassy carbon spherical powder with basal plane pyrolytic graphite electrode, ^*∗∗*^Hg(II) complex containing carbon paste electrode, ^*∗∗∗*^MWNT: multiwall carbon nanotubes, GCE: glassy carbon electrode, ^*∗∗∗∗*^GCM: glassy carbon metal, and MWCNT: multiwall carbon nanotube.
